# Interpretable machine learning model integrating clinical and elastosonographic features to detect renal fibrosis in Asian patients with chronic kidney disease

**DOI:** 10.1007/s40620-023-01878-4

**Published:** 2024-02-05

**Authors:** Ziman Chen, Yingli Wang, Michael Tin Cheung Ying, Zhongzhen Su

**Affiliations:** 1 Department of Health Technology and Informatics, The Hong Kong Polytechnic University, Kowloon, Hong Kong; 2 Ultrasound Department, EDAN Instruments, Inc., Shenzhen, China; 3 Department of Ultrasound, Fifth Affiliated Hospital of Sun Yat-Sen University, Zhuhai, China

**Keywords:** Chronic kidney disease, Renal fibrosis, Elastography, Machine learning, Shapley additive explanation

## Abstract

**Background:**

Non-invasive renal fibrosis assessment is critical for tailoring personalized decision-making and managing follow-up in patients with chronic kidney disease (CKD). We aimed to exploit machine learning algorithms using clinical and elastosonographic features to distinguish moderate-severe fibrosis from mild fibrosis among CKD patients.

**Methods:**

A total of 162 patients with CKD who underwent shear wave elastography examinations and renal biopsies at our institution were prospectively enrolled. Four classifiers using machine learning algorithms, including eXtreme Gradient Boosting (XGBoost), Support Vector Machine (SVM), Light Gradient Boosting Machine (LightGBM), and K-Nearest Neighbor (KNN), which integrated elastosonographic features and clinical characteristics, were established to differentiate moderate-severe renal fibrosis from mild forms. The area under the receiver operating characteristic curve (AUC) and average precision were employed to compare the performance of constructed models, and the SHapley Additive exPlanations (SHAP) strategy was used to visualize and interpret the model output.

**Results:**

The XGBoost model outperformed the other developed machine learning models, demonstrating optimal diagnostic performance in both the primary (AUC = 0.97, 95% confidence level (CI) 0.94–0.99; average precision = 0.97, 95% CI 0.97–0.98) and five-fold cross-validation (AUC = 0.85, 95% CI 0.73–0.98; average precision = 0.90, 95% CI 0.86–0.93) datasets. The SHAP approach provided visual interpretation for XGBoost, highlighting the features’ impact on the diagnostic process, wherein the estimated glomerular filtration rate provided the largest contribution to the model output, followed by the elastic modulus, then renal length, renal resistive index, and hypertension.

**Conclusion:**

This study proposed an XGBoost model for distinguishing moderate-severe renal fibrosis from mild forms in CKD patients, which could be used to assist clinicians in decision-making and follow-up strategies. Moreover, the SHAP algorithm makes it feasible to visualize and interpret the feature processing and diagnostic processes of the model output.

**Graphical Abstract:**

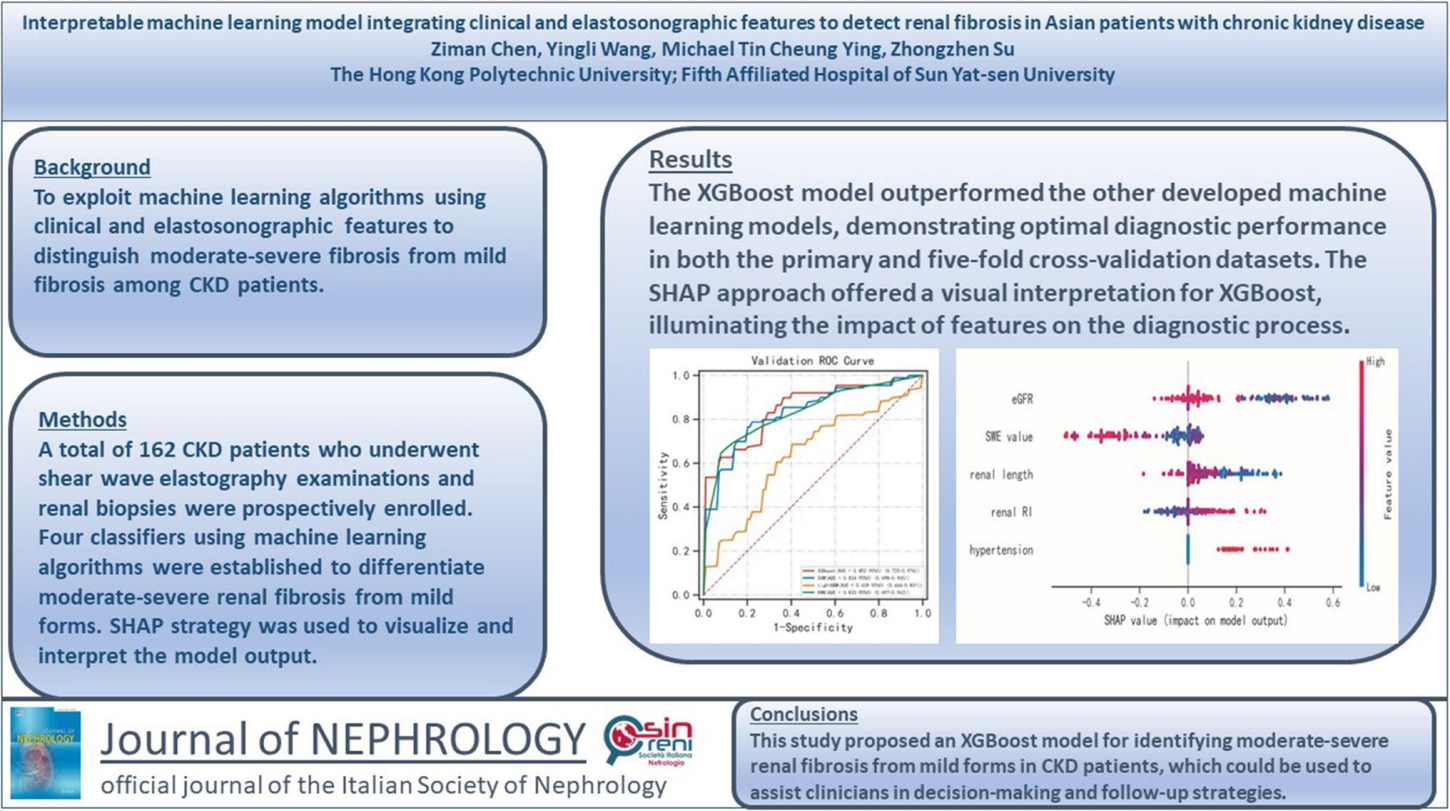

**Supplementary Information:**

The online version contains supplementary material available at 10.1007/s40620-023-01878-4.

## Introduction

In recent years, chronic kidney disease (CKD) has been well-identified as a leading global public health issue [1, 2]. Approximately 13% of people around the world are estimated to have CKD, while between 4.9 and 7.1 million people are estimated to require renal replacement therapy due to kidney failure [3]. It can be argued that CKD directly impacts the burden of morbidity and mortality among non-communicable diseases, via its effect on cardiovascular risk, at the global level. Renal fibrosis, characterized by fibrotic remodeling of the extracellular matrix, is a progressive process that deteriorates renal function in CKD [4, 5]. In fact, it represents the common final pathway in the progression of nearly all types of CKD to kidney failure, regardless of cause. An accurate diagnosis and staging of renal fibrosis are therefore prerequisites for stratifying CKD patients into distinct risk groups in order to tailor personalized therapeutic decisions based on the clinical course.

Presently, renal biopsy remains the gold standard for assessing renal fibrosis in CKD patients [6]. This method is, however, intrinsically limited by its invasive nature, which hinders its clinical application in dynamic surveillance to monitor disease progression and therapeutic response [7]. Shear wave elastography, a cutting-edge imaging technique in ultrasound (US) that can measure the elastic properties of a tissue by tracking the propagation of shear waves induced by acoustic radiation force within the target, has attracted a great deal of attention as a promising, non-invasive way to assess renal fibrosis in recent years [8, 9]. Despite this, shear wave elastography diagnostic efficacy is not yet satisfactory in routine clinical practice for this medical condition. In light of the aforementioned shortcomings, there is growing interest in exploring noninvasive approaches that may reliably evaluate renal fibrosis in CKD patients.

Machine learning is a data-driven approach derived from artificial intelligence that involves the computer identifying patterns among data sets and making decisions based on these patterns [10]. Recent decades have seen a significant increase in the application of machine learning algorithms for the analysis of critical clinical problems, leading to practical breakthroughs and research innovations [11–13]. This progress has been favored by more and more researchers in the medical field and resulted in a comprehensive set of capabilities applicable to a variety of medical conditions. However, the “black box” problem along with machine learning, which is a lack of transparency and interpretation of the decision-making process, leads to clinicians mistrusting the outcome or even ignoring recommendations altogether [14, 15].

To address the issues raised above, in this study, we intend to propose an interpretable machine learning model to assess renal fibrosis in patients with CKD. The purpose of this study was, first, to construct machine learning-based models using four distinct classifiers incorporating elastosonographic features with clinical characteristics to differentiate mild and moderate-severe renal fibrosis; second, to compare and validate the performance of the developed diagnostic models; and third, to comprehend the feature processing and decision process of the best-performing diagnostic model. To the best of our knowledge, this is the first study to propose an interpretable machine learning model integrating elastosonographic features and clinical variables to distinguish moderate-severe fibrosis from mild fibrosis in CKD patients.

## Materials and methods

### Study population

This was a cross-sectional prospective study, for which we obtained informed consent from the patients and approval from the institution’s ethical committee. Subjects who underwent renal shear wave elastography examination and renal biopsy in our department were screened for this study between April 2019 and December 2021. The inclusion criteria were the following: (1) patients diagnosed with CKD as per the Kidney Disease Improving Global Outcomes (KDIGO) 2012 guidelines [16]; (2) renal shear wave elastography examination performed before renal biopsy; (3) renal biopsy specimens graded according to the degree of fibrosis; and (4) complete laboratory evaluations for proper clinical management of the patients. The exclusion criteria were the following: (1) patients who had multiple renal cysts, renal masses, nephroliths, or hydronephrosis, or who failed to hold their breath as instructed during the examination, which affected the shear wave elastography measurements; (2) patients who were unable to undergo a successful shear wave elastography examination due to obesity or mental tension; (3) patients whose renal biopsy samples were insufficient for an assessment of fibrosis. In this study, 162 patients were ultimately enrolled as the primary dataset based on the inclusion and exclusion criteria. Laboratory biochemical indicators of each individual within seven days before renal biopsy were collected, including estimated glomerular filtration rate (eGFR), blood urea nitrogen, serum creatinine, serum uric acid, serum albumin, serum glucose, triglycerides, and urine protein to creatinine ratio, as well as medical history, including diabetes, hypertension, and cardiovascular disease.

### Shear wave elastography examination

**Fig. 1:**
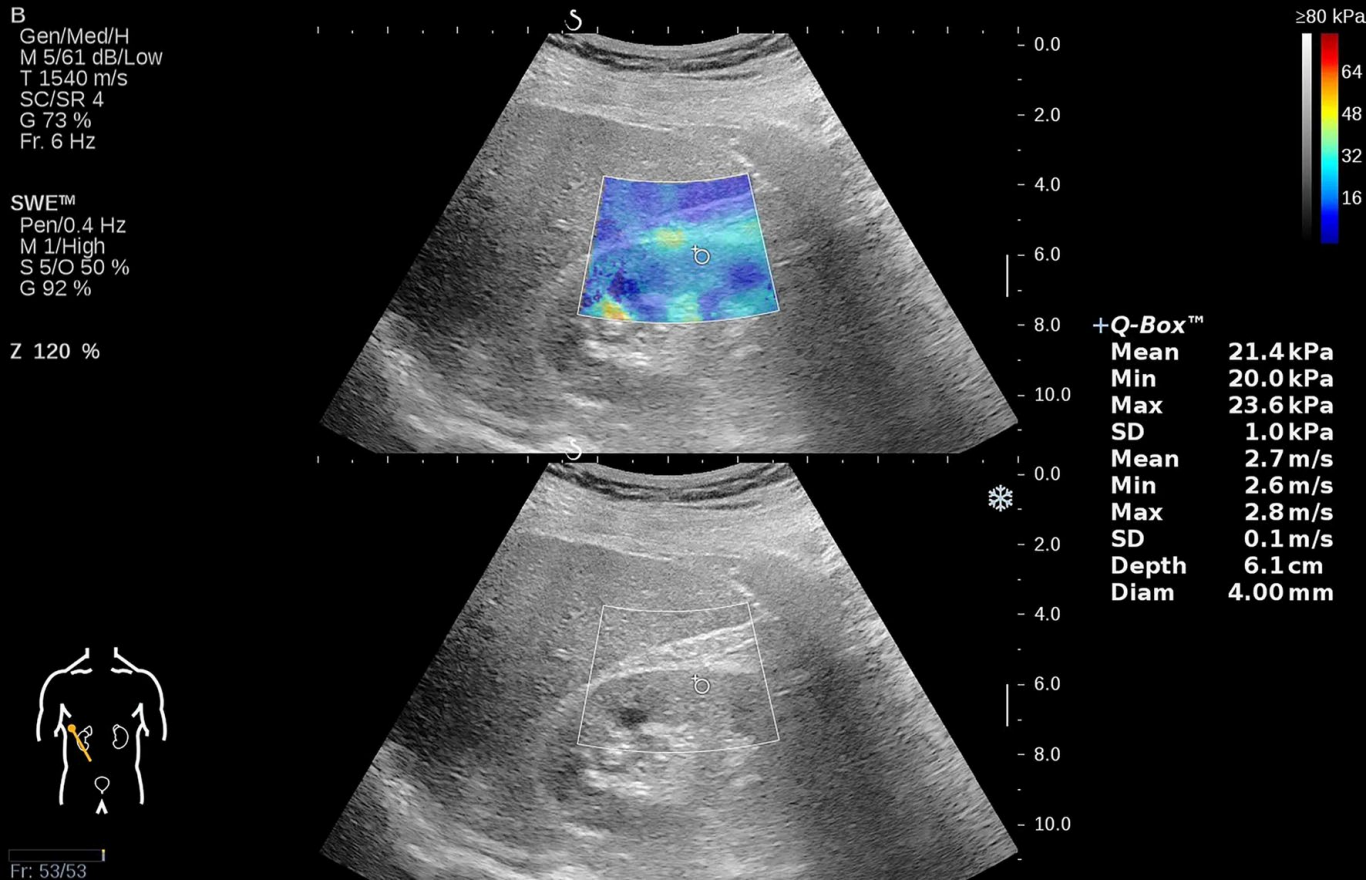
Using dual-modal display for shear wave elastography examination, the top image shows a color-coded elastogram for renal stiffness measurement, while the bottom image shows a grayscale B-mode image for guidance during real-time elasticity imaging

All renal shear wave elastography examinations were conducted by a board-certified radiologist with extensive experience in abdominal shear wave elastography within two days prior to renal biopsy using the Aixplorer US imaging system (SuperSonic Imagine, Aix-en-Provence, France) equipped with the convex array probe (SC6-1, 1–6 MHz). Patients were asked to void their bladders before examination and instructed to hold their breath for a few seconds during each measurement. On the maximum coronal section of the right kidney in a supine position, a real-time shear wave elastography procedure was performed under the guidance of B-mode US to measure the elastic modulus of the cortex in the renal middle portion, and the maximum shear wave elastography value (displayed as Emax) was recorded (Fig. 1). For each patient, five independent and valid shear wave elastography values were obtained, and the arithmetic mean was calculated to provide further analysis. Additionally, the longitudinal diameter, middle parenchyma thickness, and interlobar arterial resistive index of the right kidney were also measured and recorded. Note that our previous study demonstrated that, when compared to other shear wave elastography parameters, maximum shear wave elastography offered the highest ability to distinguish between varying degrees of renal fibrosis severity [9]. A detailed description of the examination can also be found in our previously published work.

### Renal biopsy

An US-guided percutaneous renal biopsy was conducted on the lower pole of the right kidney with a 16 or 18 G needle (Bard Magnum, Covington, GA). A series of kidney tissue specimens was stained with hematoxylin–eosin, Grocott’s methenamine silver, Masson’s trichrome, and periodic acid-Schiff and routinely examined by two dedicated pathologists using light microscopy, immunofluorescence, and electron microscopy, wherein disagreements between the two experts were resolved via discussion. Morphometric analysis of renal chronic histopathological changes was performed based on a semiquantitative scoring system described in our previous study [9]. The cases were classified into three categories based on their chronicity scoring: mild (9 points), moderate (10–18 points), and severe (19 points). Since the severe cases in this study are limited (*n* = 18), the moderately and severely impaired groups were combined to form a moderate-severe group that was then compared against the mild group in the subsequent analyses.

### Model establishment and evaluation

Using univariate and multivariate analyses, independent risk factors from elastosonographic features and clinical characteristics were identified for the differentiation between mild and moderate-severe renal fibrosis. That is to say, the variables with *P* < 0.05 in the univariate analysis (Chi-squared or Fisher’s exact tests for categorical variables and Student’s *t* test or Mann–Whitney *U* test for continuous variables, as appropriate) were entered into the multivariate logistic regression analysis to obtain significant factors (*P* < 0.05). Four different machine learning algorithms, namely eXtreme Gradient Boosting (XGBoost), Support Vector Machine (SVM), Light Gradient Boosting Machine (LightGBM), and K-Nearest Neighbor (KNN), were utilized to establish the diagnostic model in this study, respectively. For each classifier, a grid search strategy was applied to identify the optimal hyperparameter configuration [17]. Further, a five-fold cross-validation scheme was employed to verify the performance and generalization of the developed models. In brief, the primary dataset is divided into five complementary partitions, of which four-fifths are used for training models and one-fifth is used for testing. Five-folds were traversed five times as a test set. A single performance metric estimate was created by averaging five classification test results. Model performance was assessed with a receiver operating characteristic (ROC) curve and a precision-recall curve. The corresponding performance metrics were calculated, including the area under the ROC curve (AUC), sensitivity, specificity, accuracy, F1 score, and average precision (i.e., area under the precision-recall curve).

### Model interpretability in machine learning

In this study, the SHAP (SHapley Additive exPlanations) algorithm is exploited to solve the “black box” problem of machine learning [18]. A primary objective of SHAP is to explain the diagnosis of an instance by calculating the contributions of each feature to the diagnosis. As part of the SHAP explanation method, Shapley values are identified from coalitional game theory, in which the Shapley values are used to determine how to distribute the “payout” (i.e., the diagnosis) among the features fairly. Compared to other interpretability methods, SHAP is characterized by three desirable characteristics: local accuracy, consistency, and missingness. Specifically, SHAP feature importance was used for ranking features by reducing the importance of those features in relation to the average absolute Shapley values. Further, a summary plot combining feature importance with feature effects was proposed to facilitate the visualization of the relationship between feature value and diagnosis impact. To analyze diagnosis results at the individual level, a SHAP explanation force plot was developed. A feature attribution, such as Shapley value, is visualized as a force that either increases (represented by red arrows) or decreases (represented by blue arrows) the risk probability from the baseline, and these forces balance each other out when the data instance is actually diagnosed.

### Statistical analysis

All statistical analyses were performed using R version 3.6.3 and Python version 3.7. Continuous variables were presented as means ± standard deviations (SD) or medians (interquartile ranges), as appropriate, whereas categorical variables were presented as frequencies (percentages). A two-sided *P* value of < 0.05 was considered statistically significant.

## Results

### Baseline characteristics of study patients

Among the 162 CKD patients included, 74 presented with pathology-confirmed mild fibrosis, while 88 exhibited moderate-severe fibrosis. Within this patient cohort, IgA nephropathy emerged as the predominant condition (44.4%), followed by membranous nephropathy (21%) and minimal change nephropathy (9.9%). In the subgroup of patients with mild fibrosis, 70 cases (94.59%) presented with CKD stages 1–3, while 4 cases (5.41%) exhibited CKD stages 4–5. Within the subset of patients diagnosed with moderate-severe fibrosis, 76 cases (86.36%) had CKD stages 1–3, and 12 cases (13.64%) were identified with CKD stages 4–5. A univariate analysis revealed significant differences between the two renal fibrosis groups regarding age, eGFR, blood urea nitrogen, serum creatinine, renal length, renal resistive index, shear wave elastography value, and comorbidities (such as diabetes, hypertension, and cardiovascular disease). In multivariate analysis, the following five variables remained significantly associated with the study outcome and were retained for machine learning modeling: eGFR, renal length, renal resistive index, shear wave elastography value, and hypertension. In particular, compared to patients with mild fibrosis, the moderate-severe fibrosis group exhibited lower eGFR, renal length, and shear wave elastography values, as well as higher renal resistive index and hypertension proportions. Further information regarding baseline characteristics can be found in Table 1, while the etiology of CKD patients is delineated in Table S1.

**Table 1:** Baseline characteristics and feature analysis of cohort study participants by renal fibrosis categories

Characteristic	Mild fibrosis (*n* = 74)	Moderate-severe fibrosis (*n* = 88)	*P*-value	
			Univariate analysis	Multivariate analysis
Demographic information				
Age (years)	34.47 ± 12.90	45.40 ± 13.48	** < 0.001**	0.141
Sex				
Male	43 (58.11)	48 (54.55)	0.649	
Female	31 (41.89)	40 (45.45)		
BMI (kg/m^2^)	24.49 ± 4.13	23.81 ± 3.30	0.248	
Liquid biopsy indicator				
eGFR (mL/min/1.73 m^2^)	101.21 ± 28.53	67.23 ± 33.67	** < 0.001**	** < 0.001**
Blood urea nitrogen (mmol/L)	4.61 (3.60–5.59)	6.21 (5.01–7.93)	** < 0.001**	0.650
Serum creatinine (umol/L)	71.50 (56.25–92.93)	101.00 (82.50–148.00)	** < 0.001**	0.876
Serum uric acid (umol/L)	387.85 ± 105.01	398.09 ± 94.34	0.647	
Serum albumin (g/L)	31.86 ± 10.80	33.79 ± 8.13	0.255	
Serum glucose (mmol/L)	4.55 ± 0.81	4.96 ± 1.44	0.062	
Triglyceride (mmol/L)	1.62 (1.16–2.19)	1.63 (1.04–2.35)	0.851	
UPCR (g/gCr)	1.56 (0.36–6.66)	1.88 (0.61–4.56)	0.804	
Elastosonographic parameter				
Renal length (cm)	10.62 ± 0.78	10.26 ± 0.95	**0.010**	**0.025**
Parenchyma thickness (cm)	1.64 ± 0.29	1.58 ± 0.27	0.147	
RI	0.62 ± 0.05	0.65 ± 0.07	**0.006**	**0.014**
SWE value (kPa)	39.63 ± 9.26	29.96 ± 8.15	** < 0.001**	** < 0.001**
Comorbidity				
Diabetes				
Yes	4 (5.41)	14 (15.91)	**0.034**	0.540
Hypertension				
Yes	10 (13.51)	43 (48.86)	** < 0.001**	** < 0.001**
Cardiovascular disease				
Yes	2 (2.70)	12 (13.64)	**0.014**	0.821

Bold values indicate statistical significance

Continuous variables are presented as mean ± standard deviation or median (interquartile range) and categorical variables as *n* (%) as appropriate

*BMI* Body mass index; *eGFR* estimated glomerular filtration rate; *UPCR* urine protein to creatinine ratio; *RI* resistive index; *SWE* shear wave elastography

### Performance comparison of machine learning models

**Fig. 2:**
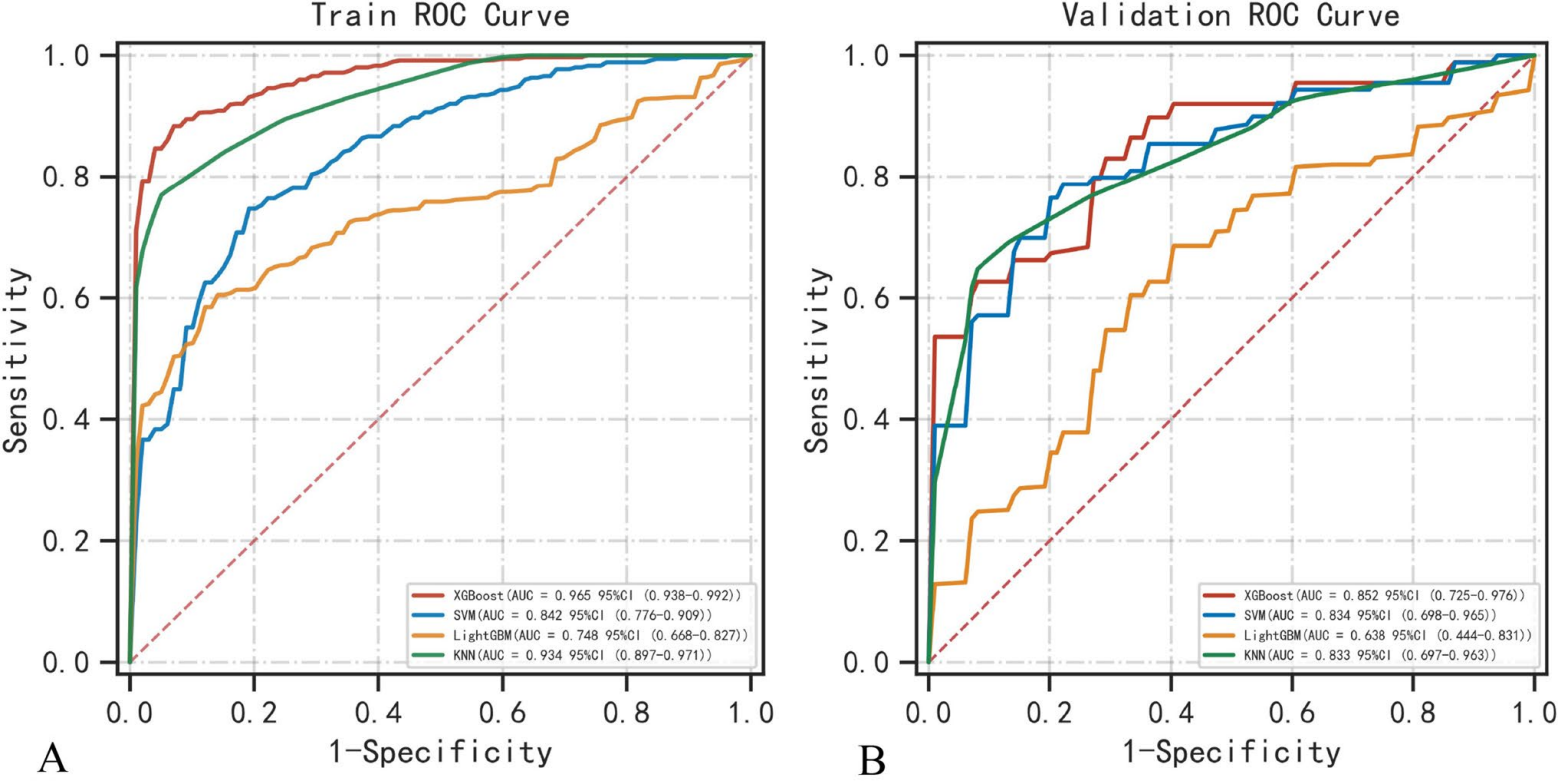
A comparison of receiver operating characteristic curves for each classifier in the primary cohort (**A**) and five-fold cross-validation cohort (**B**). The red, blue, yellow, and green curves represent XGBoost, SVM, LightGBN, and KNN, respectively. *XGBoost* extreme gradient boosting; *SVM* support vector machine; *LightGBM* light gradient boosting machine; *KNN* K-nearest neighbor

**Fig. 3:**
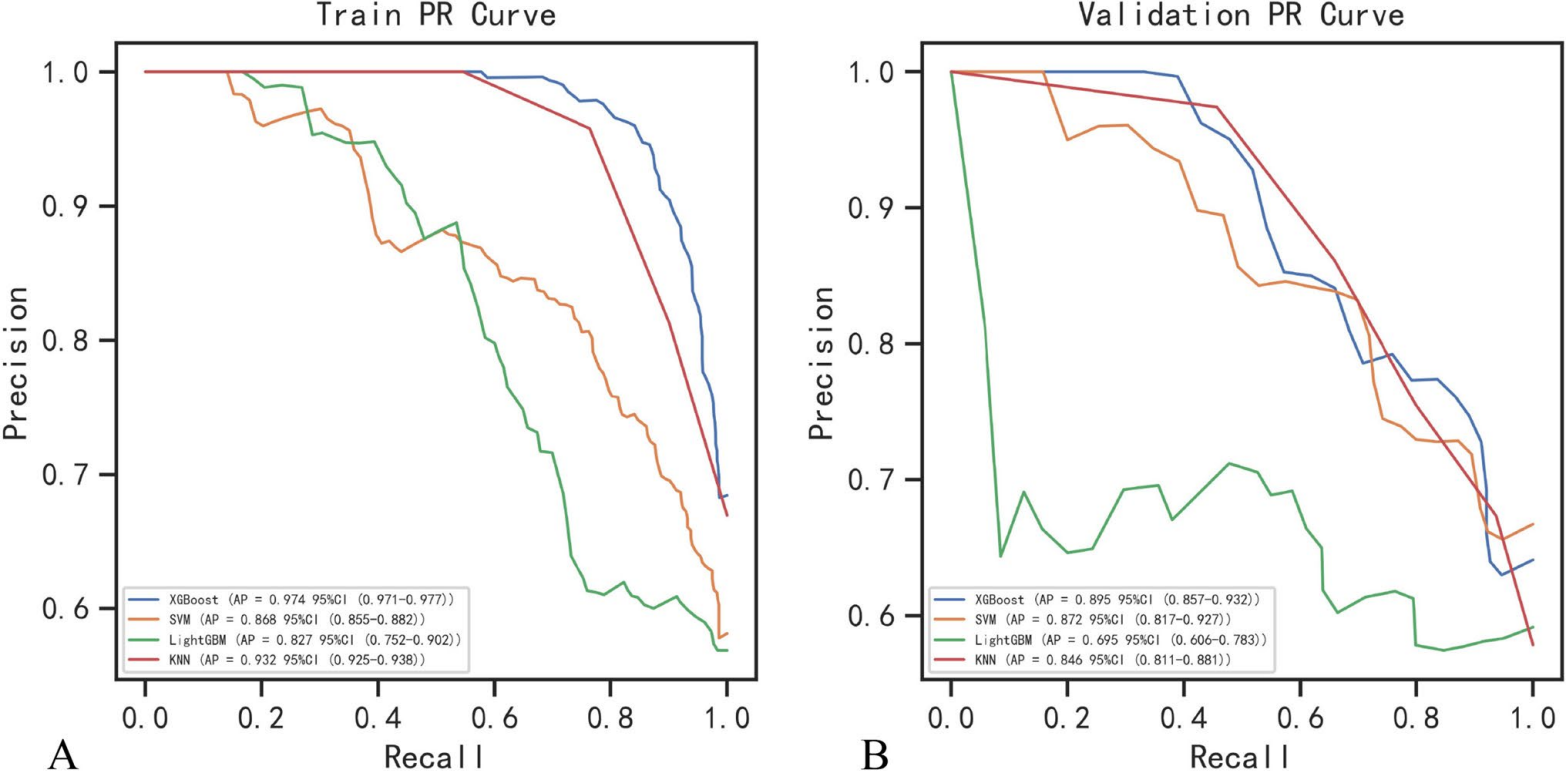
A comparison of precision-recall curves for each classifier in the primary cohort (**A**) and five-fold cross-validation cohort (**B**). The blue, yellow, green and red curves represent XGBoost, SVM, LightGBN, and KNN, respectively. *XGBoost* extreme gradient boosting; *SVM* support vector machine; *LightGBM* light gradient boosting machine; *KNN* K-nearest neighbor

In this study, four machine learning models were constructed using the aforementioned independent risk factors. As shown in Figs. 2A and 3A, optimal diagnostic performance was observed for XGBoost in the primary dataset (AUC = 0.97, 95% confidence interval (CI) 0.94–0.99; average precision = 0.97, 95% CI 0.97–0.98), followed by KNN (AUC = 0.93, 95% CI 0.90–0.97; average precision = 0.93, 95% CI 0.93–0.94), SVM (AUC = 0.84, 95% CI 0.78–0.91; average precision = 0.87, 95% CI 0.86–0.88), and LightGBM (AUC = 0.75, 95% CI 0.67–0.83; average precision = 0.83, 95% CI 0.75–0.90). Thus, XGBoost outperformed the other machine learning models in the primary cohort. Using a five-fold cross-validation analysis, the XGBoost model still achieved excellent AUC and average precision, with values of 0.85 (95% CI 0.73–0.98) and 0.90 (95% CI 0.86–0.93), which was also superior to the other three models (KNN: AUC = 0.83, 95% CI 0.70–0.96; average precision = 0.85, 95% CI 0.81–0.88; SVM: AUC = 0.83, 95% CI 0.70–0.97; average precision = 0.87, 95% CI 0.82–0.93; LightGBM: AUC = 0.64, 95% CI 0.44–0.83; average precision = 0.70, 95% CI 0.61–0.78) (Figs. 2B, 3B). The detailed performance metrics for model comparison are presented in Tables 2 and 3.

**Table 2:** Comparison of model performance in the primary cohort

Model	AUC (95% CI)	Sensitivity (95% CI)	Specificity (95% CI)	Accuracy (95% CI)	F1 score (95% CI)	AP (95% CI)
XGBoost	0.97 (0.94–0.99)	0.89 (0.87–0.90)	0.95 (0.92–0.97)	0.91 (0.89–0.92)	0.92 (0.90–0.93)	0.97 (0.97–0.98)
SVM	0.84 (0.78–0.91)	0.77 (0.71–0.83)	0.81 (0.76–0.87)	0.78 (0.77–0.79)	0.80 (0.78–0.81)	0.87 (0.86–0.88)
LightGBM	0.75 (0.67–0.83)	0.60 (0.44–0.76)	0.89 (0.83–0.96)	0.71 (0.63–0.79)	0.70 (0.59–0.80)	0.83 (0.75–0.90)
KNN	0.93 (0.90–0.97)	0.76 (0.74–0.79)	0.96 (0.95–0.97)	0.75 (0.72–0.79)	0.87 (0.85–0.88)	0.93 (0.93–0.94)

*XGBoost* Extreme gradient boosting; *SVM* support vector machine; *LightGBM* light gradient boosting machine; *KNN* K-nearest neighbor; *AP* average precision; *AUC* area under the curve; *CI* confidence level

**Table 3:** Comparison of model performance in the fivefold cross-validation

Model	AUC (95% CI)	Sensitivity (95% CI)	Specificity (95% CI)	Accuracy (95% CI)	F1 score (95% CI)	AP (95% CI)
XGBoost	0.85 (0.73–0.98)	0.84 (0.71–0.97)	0.78 (0.63–0.94)	0.73 (0.67–0.79)	0.80 (0.72–0.88)	0.90 (0.86–0.93)
SVM	0.83 (0.70–0.97)	0.80 (0.64–0.95)	0.84 (0.73–0.94)	0.73 (0.62–0.84)	0.79 (0.67–0.91)	0.87 (0.82–0.93)
LightGBM	0.64 (0.44–0.83)	0.63 (0.37–0.88)	0.73 (0.56–0.90)	0.54 (0.45–0.64)	0.60 (0.41–0.79)	0.70 (0.61–0.78)
KNN	0.83 (0.70–0.96)	0.71 (0.56–0.85)	0.89 (0.80–0.98)	0.72 (0.67–0.78)	0.80 (0.71–0.90)	0.85 (0.81–0.88)

Classifier metrics were the average of values generated using five-fold cross-validation

*XGBoost* extreme gradient boosting; *SVM* support vector machine; *LightGBM* light gradient boosting machine; *KNN* K-nearest neighbor; *AP* average precision; *AUC* area under the curve; *CI* confidence level

### Model interpretation

**Fig. 4:**
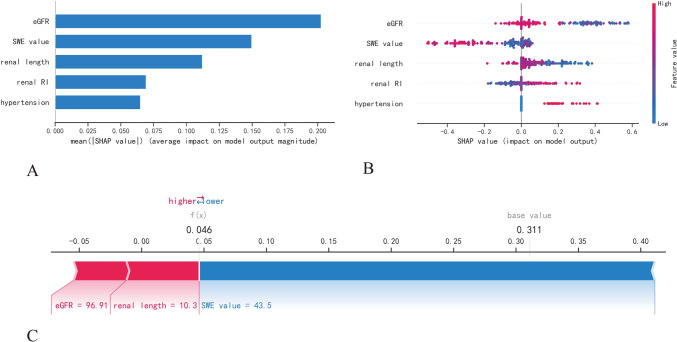
An interpretation of XGBoost based on SHAP. **A** A ranking of feature importance sorted by descending the mean absolute Shapley values of the variables. **B** SHAP summary plot of each point representing a Shapley value for a feature and an instance. The Y-axis ranks the features as per their importance to model performance, while the X-axis shows the impact of Shapley values corresponding to a particular point on the output of the model, where positive values contribute to an increase in risk and negative values contribute to a decrease in risk. The color spectrum from bright blue to bright red indicates feature values from low to high. **C** An interpretable example of a SHAP explanation force plot for a CKD patient with a predicted mild fibrosis probability that was confirmed by renal pathology. Each Shapley value on the plot represents a force pushing to increase (red arrow) or decrease (blue arrow) the prediction, which ultimately balances out. *SHAP* shapley additive explanations; *XGBoost* extreme gradient boosting; *eGFR* estimated glomerular filtration rate; SWE, shear wave elastography; *RI* resistive index

According to the above results, XGBoost was the most effective classification model in distinguishing moderate-severe renal fibrosis from mild forms, and thus it was deemed the best diagnostic model in this study. Then, the SHAP algorithm was applied to visualize the feature processing and diagnostic processes of XGBoost. The impact of each variable on the model output was evaluated by the SHAP feature importance plot (Fig. 4A), which indicated that eGFR made the largest contribution to the diagnostic model, followed by the shear wave elastography value, then renal length, renal resistive index, and hypertension. In particular, as shown in the SHAP summary plot (Fig. 4B), the lower the eGFR, the higher the Shapley value, and the greater the likelihood of moderate-severe renal fibrosis, while the same trend was observed for the shear wave elastography value. Refer to the details in Fig. 4B’s legend. A clinical case example is presented in Fig. 4C to illustrate the diagnostic process of XGBoost using the SHAP explanation force plot. The risk information for this CKD patient is the result of two opposing forces coming to a balance, in which the risk-decreasing effect derived from the shear wave elastography value is offset by the risk-increasing effect derived from eGFR and renal length. Finally, this subject obtained a low-risk probability of 4.6%, with the corresponding model output being mild renal fibrosis, which was supported by renal pathology.

## Discussion

In the current study, four machine learning models combining elastosonographic features and clinical variables were developed to discriminate between mild and moderate-severe fibrosis in CKD patients. The XGBoost model exhibited optimal diagnostic capability, which could serve as an effective and reliable noninvasive tool for clinical decision-making relating to CKD patients. As determined by the SHAP algorithm, eGFR contributed the most to the XGBoost model. In addition, the SHAP approach was also used to visualize and interpret the diagnostic process of the XGBoost model at the individual level.

As data processing technology develops, machine learning is increasingly being introduced into the domain of medicine to support personalized clinical decisions [19, 20]. In fact, there have been several studies that applied machine learning to evaluate renal fibrosis or kidney disease status. Zhu et al. exploited a SVM model that combined the shear wave elastography value with traditional US features to differentiate the severity of tubulointerstitial fibrosis among CKD patients and obtained AUC values between 0.64 and 0.94 [21]. However, they did not compare the performance of multiple machine learning models with respect to this medical issue. A study by Li et al. constructed and compared several machine learning models based on US parameters to diagnose renal disease, yielding AUC values ranging from 0.83 to 0.91 [22]. Nevertheless, the assessment of the models’ performance in that study was inadequate, as none of the models underwent internal or external validation, so their generalizability is unknown. Last but not least, even though these studies led to progress, they only looked at how well the model performed. The model’s output, however, lacks transparency, interpretability, and a clear understanding of risk, making it difficult to implement in clinical practice [15, 23].

Four distinct machine learning models were established in this study, of which the XGBoost model achieved the optimal discrimination ability when compared to the others (SVM, LightGBM, and KNN), yielding an AUC of 0.97 (95% CI 0.94–0.99), average precision of 0.97 (95% CI 0.97–0.98) in the primary dataset, and an AUC of 0.85 (95% CI 0.73–0.98), average precision of 0.90 (95% CI 0.86–0.93) in the five-fold cross-validation cohort. XGBoost is a scalable end-to-end tree boosting algorithm proposed by Chen et al. [24], in which multi-classification and regression trees are used to learn nonlinear relationships between input variables and outcomes in a boosting ensemble manner, capturing and learning nonlinear and complex relations accurately [25]. In addition to being highly efficient, flexible, and portable, it also provides more accurate output and effectively prevents overfitting [26]. This makes the XGBoost algorithm suitable for use in critical medical research and has been successfully applied in some complex clinical situations. Shi et al. applied the US-based radiomics XGBoost model to evaluate the risk of central cervical lymph node metastasis in patients with papillary thyroid carcinoma and attained a satisfactory AUC of 0.91 and 0.90 in the training and test cohorts, respectively, which outperformed the other six machine learning classifiers and an experienced radiologist [27]. A study conducted by Zhang et al. revealed that, among the 10 constructed machine learning models, XGBoost had the superior comprehensive diagnostic performance for predicting sentinel lymph node metastasis, yielding an AUC of 0.95 in the training cohort and 0.91 in the validation cohort [28]. Consistent with the findings stated above, in the present study, XGBoost was superior to the other classifiers using machine learning algorithms in distinguishing moderate-severe fibrosis from mild forms in CKD patients, providing further evidence of the diagnostic capability and robustness of the proposed algorithm regarding clinical application.

During the progression of CKD, it is crucial to underscore the significance of adopting differentiated clinical decisions and treatment strategies tailored to the distinct stages of renal fibrosis [29, 30]. The application of the proposed machine learning model facilitates the prompt identification of CKD patients presenting with mild fibrosis, thereby enabling the avoidance of aggravating factors in the initial phases of the ailment. Consequently, this affords an opportunity for early interventions, mitigating the risk of further fibrotic progression. In instances where the machine learning model identifies CKD patients with moderate-severe fibrosis, an imperative shift towards a more proactive treatment paradigm becomes warranted. This approach is designed to prevent the onset of complications, defer the initiation of dialysis treatment, and enhance the overall quality of survival. Moreover, the deployment of the developed machine learning model facilitates a non-invasive, dynamic evaluation of renal fibrosis extent during CKD treatment or follow-up. This functionality enables judicious modifications to the treatment regimen, optimizing treatment efficacy.

Following a comprehensive set of univariate and multivariate analyses, five pivotal risk factors associated with the outcome event were identified from an initial pool of 18 potential candidate variables. These crucial factors include shear wave elastography value, renal length, renal resistive index, hypertension, and eGFR. Utilizing shear wave elastography, an advanced non-invasive imaging modality, enables the quantitative evaluation of tissue elastic properties through monitoring shear wave propagation induced by acoustic radiation force impulse excitation within a specified target. Previous studies have successfully highlighted the clinical efficacy of shear wave elastography in assessing renal fibrosis [8, 9, 31]. The progression of pathological changes within the renal system is marked by a noticeable decrease in kidney size, notably accentuated by a discernible reduction in renal length [32]. With the progression of renal pathological impairment, discernible alterations in the physical characteristics of the kidneys become apparent. These observable changes in kidney morphology serve as external indicators of evolving pathological processes affecting renal tissues. Fundamental processes contributing to CKD evolution involve alterations in renal microvascular perfusion. Elevated intrarenal resistive index, indicative of renal arteriolar sclerosis, correlates with advancing renal dysfunction and fibrosis [33]. Hypertension plays a critical role in both instigating and advancing renal capillary rarefaction, influencing the intricate vascular network of the kidneys and leading to a reduction in blood vessel density [34–36]. This disruption in vascular density disturbs the oxygen supply balance, exacerbating hypoxic conditions. Consequently, this sequence, initiated by hypertension, emerges as a significant driving force behind the intricate series of events contributing to CKD progression. While the precise mechanism by which hypertension triggers renal capillary rarefaction remains elusive, hypoxia-induced processes within renal capillaries, including cell atrophy and apoptosis, contribute to the progression of glomerular sclerosis, renal arteriolar sclerosis, and renal tubulointerstitial fibrosis. Within the domain of liquid biopsy indicators, eGFR emerged as a universally embraced and applied marker in medical settings for the assessment of CKD progression [16]. Nevertheless, none of the alternative liquid biopsy markers passed scrutiny in multivariate analysis. While several other liquid biopsy indices signal the onset and progression of CKD or renal fibrosis, their limitations encompass potential non-specificity to organs, exclusive association with inflammatory states or impaired organ function, and a specific inability to distinctly delineate fibrosis stages [37, 38]. Furthermore, the clinical significance of eGFR intersects with that of other liquid biopsy markers. Owing to its heightened clinical significance, eGFR assumes a robust role as a surrogate that efficaciously supplants alternative liquid biopsy indicators.

A prior study employed a multilayer perceptron classifier to evaluate renal fibrosis severity by integrating 16 clinical variables, resulting in satisfactory diagnostic accuracy [39]. As a fundamental neural network, the multilayer perceptron classifier exhibits exceptional nonlinear data processing abilities [40]. Its efficacy lies in adeptly managing a substantial volume of input variables and mapping them into a higher-dimensional feature space, autonomously assigning variable weights throughout the entire training process. With an increase in input variables, the algorithm captures more valuable information, enhancing output accuracy. However, a higher quantity of input variables necessitates more neurons for feature extraction, leading to an increase in model parameters. This expansion presents challenges to convergence, resulting in prolonged training times and potential issues such as gradient explosion. Additionally, while excelling at feature extraction from relatively large datasets, the multilayer perceptron classifier tends to overfit with smaller sample sizes, reducing its generalization performance and practical applicability. Despite its input handling advantages, careful consideration is essential due to parameter escalation and potential training challenges. Moreover, incorporating additional input variables like demographic data, laboratory indicators, and imaging parameters may improve multilayer perceptron classifier predictions but raise model application costs. The multilayer perceptron classifier built using screened independent variables in this study yielded AUCs of 0.73 (95% CI 0.64–0.83) and 0.72 (95% CI 0.54–0.89) in the training and validation sets, respectively, indicating barely satisfactory diagnostic performance in this scenario (Table S2). This investigation utilized diverse machine learning algorithms, such as XGBoost, SVM, KNN, and LightGBM, to tackle the clinical issue. The modeling parameters of these classifiers prove relatively straightforward and comprehensible. Not only do they demand a minimal set of variables for constructing models that achieve decent predictive accuracy, but they also exhibit efficiency and adaptability in practical use. XGBoost classifier is esteemed for its ensemble learning capability and remarkable performance, delivering reliable predictions even in sub-optimal feature engineering scenarios [41]. The SVM classifier excels at handling nonlinear and high-dimensional data, exhibiting superior classification accuracy for small-scale datasets [42]. The KNN classifier, known for its simplicity and intuitive nature, operates without assumptions about data distribution, proving versatile across various data types while effectively managing nonlinear data [43]. The LightGBM classifier is preferred as a gradient enhancement framework due to its efficient training speed [44]. The lightweight design of these algorithms and their minimal variable requirements significantly contribute to faster training and reduced computational costs in practical applications. This aspect holds particular significance in clinical settings characterized by limited computational resources or real-time processing

It should be noted that when using a machine learning algorithm to solve a crucial clinical problem, the “black box” problem of the model should be brought into the spotlight and addressed [14]. This means that the model’s decision-making process should be transparent and explainable instead of solely obtaining more accurate results. In this case, a SHAP strategy was introduced to demonstrate the importance and impact of features on the XGBoost model’s output and provide individual patients with a visual interpretation of their diagnostic results. As illustrated in the SHAP plot, the variable having the greatest impact on model output was eGFR, with lower eGFR values corresponding to higher Shapley values, driving an increased chance of model output being moderate-severe renal fibrosis. This finding of the SHAP algorithm was in line with what was seen in clinical practice, as a decline in kidney function was a warning sign that renal fibrosis would be exacerbated in CKD patients [45, 46]. Additionally, the SHAP algorithm revealed that, as the feature contributing the second highest amount to model output, a higher shear wave elastography value corresponding to a lower Shapley value reduced the likelihood of developing moderate-severe renal fibrosis, which was consistent with previous research [8, 9, 47]. Consequently, SHAP addresses the “black box” issue that has hindered the development of complex models by providing a personalized and reasonable explanation for diagnosis, significantly improving the application value of clinical models and clinicians’ confidence in established models.

Despite several strengths of this study, there are still some aspects worth noting. First, previous studies have identified age as an independent risk factor in renal fibrosis progression [48, 49], which aligns with the findings from the univariate analysis conducted in this study. However, the multivariate analysis did not include age as an independent variable. Taking into account the pathophysiological impact of age on shear wave elastography-measured elasticity, eGFR, renal length, and hypertension, their simultaneous incorporation into the multivariate analysis might have led to overlapping and intertwining information [50–52]. While the multivariate analysis retained shear wave elastography value, eGFR, renal length, and hypertension—each impacted by age—, it chose to exclude age itself as an independent variable. This exclusion could be attributed to these variables already capturing the diagnostic significance associated with age, thereby rendering a separate consideration of the age variable unnecessary. Second, elastography in assessing renal fibrosis remains controversial in clinical practice. Studies by Leong et al. and Yang et al. revealed an increase in shear wave elastography-measured renal stiffness corresponding to the progression of chronic renal damage characterized by glomerular sclerosis, interstitial fibrosis, and tubular atrophy [53, 54]. In contrast, our previous investigation revealed a decrease in shear wave elastography-derived elastic values as pathological damage progressed in renal fibrosis [9]. Another study conducted by Güven et al. utilizing magnetic resonance elastography to assess renal fibrosis also concluded that magnetic resonance elastography-derived stiffness values decreased in patients with chronic injury, specifically noting reduced stiffness as glomerulosclerosis and tubulointerstitial fibrosis progressed [55]. It is important to emphasize that previous studies have exhibited deficiencies in the way they have conducted their experiments, resulting in conclusions that differ from those reached by our study and that of Güven et al. For example, Leong et al.’s study utilized point-shear wave elastography for detecting renal fibrosis, lacking an elastogram during image acquisition, which hindered artifact-free region identification. Furthermore, point- shear wave elastography employed a fixed size for the region of interest, potentially leading to inaccuracies in placement and increased measurement variability by not excluding the renal medulla. In Yang et al.’s study, shear wave elastography values were obtained from the kidney’s inferior pole. Conversely, Lin et al.’s research highlighted notably lower variability coefficients in the mid-region compared to the lower pole, suggesting constrained reproducibility in measurements taken from the renal poles [31]. In order to improve reproducibility, it is recommended to refrain from measuring renal poles [56]. Another study by Leong et al. emphasized the importance of these factors on shear wave elastography assessment in renal fibrosis, suggesting that they could lead to inaccurate results and, therefore, erroneous conclusions [57]. Third, input variables, such as shear wave elastography value, renal resistive index, and hypertension, collectively indicate the influence of renal perfusion to some extent and could potentially introduce biases. Machine learning algorithms do not exclusively focus on direct associations between these variables. Instead, they are trained to manage multivariate feature coupling, aiming at precise predictions [58]. These algorithms process data by emphasizing collective effects among features, rather than concentrating solely on simple relationships. By conducting comprehensive analyses and processing multiple features, these algorithms adeptly capture and leverage intricate interactions between features to enhance predictive capabilities. Their primary objective is to refine prediction accuracy by thoroughly considering the complexity of multiple features, thereby offering a more precise understanding of data patterns and trends.

This study has some limitations. First, as the number of patients enrolled in the present study is still relatively small, future studies with a large population-based cohort, which allows more detailed analyses, are warranted. Second, considering that the current study is derived from a single center cohort, further large-scale, multicenter studies are required to validate the present findings.

## Conclusions

The proposed XGBoost model, which combines elastosonographic parameters and clinical features, demonstrated high discriminatory performance and outperformed other machine learning models in distinguishing moderate-severe renal fibrosis from mild forms in CKD patients. The SHAP algorithm visualizes and interprets the XGBoost model’s feature processing and diagnostic processes. This interpretable XGBoost model could be used to assist clinicians in critical decision-making and follow-up strategies related to renal fibrosis severity in CKD patients.

### Supplementary Information

Below is the link to the electronic supplementary material.

Supplementary file1 (DOCX 20 KB)

## Data Availability

The data presented in this study are available on reasonable request from the corresponding author. The data are not publicly available due to ethical concerns regarding privacy.
