# Supplementary material for: Interpretable machine learning model integrating clinical and elastosonographic features to detect renal fibrosis in Asian patients with chronic kidney disease
Source: J Nephrol. 2024 Feb 5;37(4):1027–39. doi: 10.1007/s40620-023-01878-4 (PMC11239734; doi:10.1007/s40620-023-01878-4)
Supplement: Supplementary file 1 — Supplementary file1 (DOCX 20 KB) [file 40620_2023_1878_MOESM1_ESM.docx]

Table S1. Etiology of CKD

| Diagnosis | Number |
| --- | --- |
| IgA nephropathy  Membranous nephropathy  Minimal change nephropathy  Mesangial proliferative glomerulonephritis  Lupus nephritis  Focal segmental glomerular sclerosis  Diabetic nephropathy  Others  Unknowns | 72 (44.4%)  34 (21.0%)  16 (9.9%)  9 (5.6%)  9 (5.6%)  8 (4.9%)  6 (3.7%)  5 (3.0%)  3 (1.9%) |

Notes: Categorical variables are presented as n (%).

Abbreviations: CKD, chronic kidney disease.

Table S2. Diagnostic performance of the MLP model

| Index | Primary cohort | | | | Cross-validation cohort | | | | |  |
| --- | --- | --- | --- | --- | --- | --- | --- | --- | --- | --- |
|  | AUC  (95% CI) | Sensitivity  (95% CI) | Specificity  (95% CI) | Accuracy  (95% CI) | | AUC  (95% CI) | Sensitivity  (95% CI) | Specificity  (95% CI) | Accuracy  (95% CI) | |
| MLP | 0.73  (0.64-0.83) | 0.70  (0.61-0.79) | 0.72  (0.52-0.92) | 0.70  (0.64-0.76) | | 0.72  (0.54-0.89) | 0.83  (0.72-0.94) | 0.64  (0.45-0.83) | 0.69  (0.57-0.80) | |

Abbreviations: MLP, multilayer perceptron; AUC, area under the curve; CI, confidence level.
